# Autonomic Non-Responsiveness in HRV Biofeedback: A Narrative Conceptual Review and Future Directions for AI-Guided Closed-Loop Adaptive Systems

**DOI:** 10.3390/medicina62061102

**Published:** 2026-06-05

**Authors:** Alexandru Burlacu, Crischentian Brinza, Adrian Iftene, Roxana-Elena Bogdan-Goroftei, Oana Geman

**Affiliations:** 1 Faculty of Medicine, University of Medicine and Pharmacy “Grigore T Popa”, 700115 Iasi, Romania; alexandru.burlacu@umfiasi.ro; 2 Institute of Cardiovascular Diseases “Prof. Dr. George I.M. Georgescu”, 700503 Iasi, Romania; 3 Computer Science Department, Faculty of Informatics, Alexandru Ioan Cuza University, 700506 Iasi, Romania; adiftene@info.uaic.ro; 4 Faculty of Medicine, “Dunarea de Jos” University, 800010 Galati, Romania; roxana.goroftei@ugal.ro; 5 Division of Data Science and Artificial Intelligence, Computer Science and Engineering, Chalmers University of Technology, 412 96 Gothenburg, Sweden; geman@chalmers.se; 6 Division of Data Science and Artificial Intelligence, Computer Science and Engineering, University of Gothenburg, 405 30 Gothenburg, Sweden

**Keywords:** heart rate variability, HRV biofeedback, autonomic regulation, autonomic non-responsiveness, resonance breathing, baroreflex, closed-loop biofeedback, artificial intelligence, physiological adaptability, personalized intervention

## Abstract

Heart rate variability (HRV) is widely used as a non-invasive marker of autonomic regulation and physiological adaptability, with relevance across cardiovascular, metabolic, neuropsychiatric, and stress-related conditions. HRV biofeedback has emerged as a non-pharmacological intervention intended to influence autonomic function through paced breathing, resonance-frequency training, and real-time physiological feedback. Although this approach has shown promise in improving stress regulation, emotional symptoms, autonomic balance, and selected cardiovascular outcomes, its effects are not consistent across individuals or clinical states. The reasons for this variability remain insufficiently conceptualized. In this narrative conceptual review, we propose the concept of autonomic non-responsiveness during HRV biofeedback as a descriptive framework for situations in which expected autonomic engagement is weakened, absent, or fails to translate into meaningful physiological or clinical benefit. We discuss potential contributors to non-response, including reduced autonomic flexibility, impaired baroreflex function, disease burden, fatigue, stress-related overload, dysfunctional breathing, methodological limitations, and cognitive-behavioral constraints. We then consider the clinical implications of recognizing non-response as a potentially informative state rather than a simple negative outcome. Finally, we outline a future research agenda focused on operational definition, candidate biomarkers, temporal characterization, and minimally adaptive closed-loop systems.

## 1. Introduction

More than 30 years ago, a landmark consensus statement helped establish heart rate variability (HRV) as a standardized framework for quantifying beat-to-beat fluctuations in sinus rhythm and interpreting their physiological meaning. Since then, HRV has become a widely used non-invasive marker of autonomic regulation, offering an indirect window into the dynamic interplay between sympathetic and parasympathetic influences on the cardiovascular system. In contemporary cardiovascular and translational research, HRV is valued not only as a descriptive signal, but also as an indicator of physiological flexibility, neurocardiac regulation, and adaptive capacity under internal and external stress [1].

The relevance of HRV extends well beyond methodological interest. Altered HRV has been associated with adverse prognosis in cardiovascular disease, including higher risks of mortality and arrhythmic or ischemic events, while also showing relevance across broader clinical contexts marked by impaired autonomic balance, stress dysregulation, inflammation, and reduced physiological resilience. For this reason, HRV is increasingly viewed not merely as a cardiac metric, but as a transdiagnostic digital biomarker of autonomic adaptability and systemic vulnerability [2].

In this context, HRV biofeedback has emerged as a non-pharmacological strategy aimed at modulating autonomic self-regulation through guided breathing and real-time physiological feedback. Most commonly, it relies on slow breathing near resonance frequency, with the goal of amplifying respiratory sinus arrhythmia, engaging baroreflex mechanisms, and improving the coordination between respiratory and cardiovascular oscillations. In this sense, HRV biofeedback is not simply an attempt to “increase HRV” in isolation, but rather to influence autonomic regulation more broadly, with HRV serving as a measurable marker of that physiological engagement [3].

The rationale for HRV biofeedback is compelling, and systematic reviews support its potential to improve emotional and physical health. However, its benefits are not uniform across individuals or across clinical states. Methodological heterogeneity remains substantial, and some individuals appear unable to achieve the expected autonomic pattern during training or fail to derive meaningful physiological or clinical benefit despite apparently adequate exposure. This recurring variability suggests that non-response should not be dismissed as a trivial issue of compliance alone [4].

Earlier biofeedback research also recognized that the acquisition of voluntary cardiovascular control is not uniform. Some studies described feedback-based attempts to decrease or increase heart rate and emphasized that success depends on the individual’s capacity to develop and stabilize new regulatory responses under feedback conditions. These data suggests that failure to achieve the intended autonomic pattern may reflect limits in physiological self-regulation, learning, or person–protocol fit, rather than merely poor adherence or inadequate effort [5,6].

In this article, we suggest that part of the uneven response to HRV biofeedback [7] may be understood through the concept of autonomic non-responsiveness during HRV biofeedback. We use the term autonomic non-responsiveness during HRV biofeedback to describe situations in which the expected autonomic engagement is weakened, absent, or does not lead to meaningful physiological or clinical benefit. We do not present this as a fundamentally new physiological discovery or as a formal diagnostic entity, but as a conceptual framework that may help make sense of a familiar yet insufficiently examined pattern. On this basis, we discuss potential mechanisms of non-response, consider its clinical implications, and outline how future adaptive, possibly AI-guided, approaches may help identify and address this phenomenon more precisely.

This article is a narrative review. The literature was selected to support conceptual development rather than to provide a systematic evidence synthesis. We prioritized foundational HRV standards, HRV biofeedback mechanisms and guidelines, studies addressing responder heterogeneity or training proficiency, and recent literature on adaptive biofeedback, wearable monitoring, and AI-based physiological signal interpretation.

## 2. HRV, Autonomic Regulation, and Why HRV Biofeedback Matters

### 2.1. HRV as a Biomarker of Autonomic Regulation

Reduced HRV often reflects impaired vagal modulation or autonomic imbalance, but the physiological meaning of individual HRV metrics depends on the recording conditions and on the specific index being analyzed. Nevertheless, both experimental and conceptual work has shown that low-frequency HRV and the LF/HF ratio should not be treated as direct markers of sympathetic activity or “sympatho-vagal balance” [8,9]. Accordingly, HRV is better viewed as an indirect and context-sensitive marker of autonomic influence on the heart, one that captures the adaptive responsiveness of a complex control system rather than the isolated output of one autonomic branch [1,10].

From a broader physiological perspective, HRV is clinically attractive because it reflects flexibility within the organism’s regulatory mechanisms. Neurovisceral models have linked higher resting vagally mediated HRV with more adaptive integration of prefrontal, affective, and autonomic processes, supporting self-regulation and more efficient adaptation to environmental demands [11]. Although this framework should not be overstated, it offers a useful rationale for understanding why reduced HRV frequently accompanies conditions marked by autonomic rigidity, impaired recovery, and diminished physiological flexibility [11].

### 2.2. HRV Across Disease States: Prognostic Relevance

The clinical importance of HRV first became evident in cardiovascular disorders, where reduced HRV displayed prognostic value beyond conventional risk markers. In the Framingham Heart Study, lower HRV independently predicted incident cardiac events in participants without clinically apparent coronary heart disease or heart failure [12]. This association has since been reinforced at the pooled level. In a meta-analysis of populations without known cardiovascular disease, low HRV was associated with a 32% to 45% higher risk of a first cardiovascular event [13]. In patients with established cardiovascular disease, lower HRV has likewise been associated with a higher risk of all-cause death and future cardiovascular events [13,14,14].

Moreover, reduced HRV has also been reported in chronic kidney disease, where it predicts mortality and highlights autonomic dysfunction in a population with high cardiovascular risk [15]. Similar observations have been reported in diabetes, where autonomic impairment reflected by reduced HRV is linked to adverse outcomes and mortality risk [16]. Taken together, these findings suggest that HRV is not a disease-specific marker, but rather a signal of altered autonomic regulation that becomes clinically meaningful in different ways across disease states [17].

### 2.3. The Importance of HRV Biofeedback

Thus, HRV biofeedback is important because it represents one of the few non-pharmacological interventions explicitly designed to modulate autonomic regulation in real time. In its classic form, the patient is guided to breathe slowly, usually near an individually determined resonance frequency, while receiving beat-to-beat physiological feedback derived from the cardiovascular signal [3,18,19,20]. The intervention is therefore not aimed at “raising HRV” as an isolated numerical goal. Rather, it seeks to influence the autonomic system itself, with HRV serving as a measurable marker of how that system is responding [21].

The physiological rationale for HRV biofeedback is relatively consistent across the literature. Breathing near resonance frequency, often close to 0.1 Hz in many adults, amplifies respiratory sinus arrhythmia, engages resonance properties of the cardiovascular system, and promotes stronger baroreflex oscillation and coupling between breathing, blood pressure, and heart period dynamics [7,21]. Experimental work by Lehrer and colleagues showed that HRV biofeedback can increase baroreflex gain, supporting the idea that the intervention is not merely relaxing, but capable of training a relevant autonomic reflex pathway [19]. More recent reviews and methodological guidelines have also emphasized that resonance frequency is individual rather than uniform, which helps explain why fixed breathing prescriptions may produce inconsistent results across participants.

Therefore, the key point is not that HRV biofeedback works uniformly in every individual or in every condition. Rather, its relevance lies in the fact that it directly targets autonomic regulation. The challenge, however, is that the expected autonomic engagement does not appear to occur consistently across all individuals and states. That observation provides the rationale for the present conceptual discussion of autonomic non-responsiveness during HRV biofeedback [7].

## 3. Framing the Clinical Problem: Autonomic Non-Responsiveness During HRV Biofeedback

### 3.1. Not Everyone Responds

Although HRV biofeedback has demonstrated therapeutic promise across a wide range of settings, its effects are not uniform. Foundational work has clarified plausible mechanisms for HRV biofeedback, and systematic review data support its benefits across multiple domains of emotional, physical, and performance-related functioning. The literature also suggests substantial heterogeneity in how HRV biofeedback is applied and in the degree to which individuals appear to benefit from it. In this sense, the field already contains the observation, even if not yet a unified concept, that expected physiological and clinical gains are not achieved consistently across individuals [3,4].

This variability is unlikely to be trivial. A methodological review has noted that there is still no consensus on how HRV biofeedback should be applied, including differences in resonance-frequency assessment, pacing strategy, protocol design, and outcome reporting [7]. In parallel, computational physiology work has suggested that individuals may differ not only in the magnitude but also in the nature of cardiovascular benefit during resonance breathing, supporting the idea that responsiveness itself may be heterogeneous and that intervention tailoring may be clinically relevant [22].

Taken together, these findings indicate that non-uniform response should not be reduced to a simple issue of compliance or motivation.

Importantly, the literature already contains pragmatic signals of this problem. Courtney and colleagues reported that dysfunctional breathing patterns were associated with reduced ability to achieve target HRV patterns during biofeedback, suggesting that breathing assessment may help identify poor responders who require greater emphasis on breathing correction [23]. More recently, Saito and colleagues showed that individuals with higher resting vagally mediated HRV were more likely to be proficient in HRV biofeedback training and to benefit from its anxiety-reducing effects, noting that such findings may inform participant selection and the modification of training protocols for non-responders [24].

Thus, while the HRV biofeedback literature does not appear to formally recognize “autonomic non-responsiveness” as an established construct, it does point repeatedly to poor responder profiles, reduced benefit in some individuals, and participant characteristics that may shape training efficacy [23].

These observations point to the need for a more explicit conceptual framework. Without one, markedly different situations may be grouped together under vague labels such as limited efficacy, poor adherence, inadequate technique, or negative study findings, even when the underlying problem may instead reflect differences in physiological readiness, autonomic reserve, breathing pattern integrity, or person–protocol fit. What is needed is not a new diagnosis, but a clearer way of naming and organizing a recurring clinical and experimental observation [7].

### 3.2. Defining the Proposed Construct

In this context, autonomic non-responsiveness may be conceptualized as a transient or persistent state in which expected autonomic engagement fails to occur, is attenuated, or does not translate into meaningful physiological or clinical benefit.

We introduce this term as a descriptive and explanatory construct grounded in the recurrent observation that HRV biofeedback does not elicit comparable autonomic engagement across individuals or states [4]. Framed in this way, the concept is intended to unify an under-discussed pattern that has been suggested fragmentarily in the literature.

This construct can be understood across at least three interconnected levels.

First, autonomic non-responsiveness may describe an absent or blunted immediate physiological response during the biofeedback process itself. In such cases, the expected autonomic pattern—such as adequate cardiorespiratory coupling, coherent oscillatory response, or a robust HRV shift during paced breathing—fails to emerge in a convincing way. This level is consistent with evidence that resonance-based responses and cardiovascular effects can vary meaningfully between individuals and may depend on protocol characteristics as well as physiological state [22].

Second, autonomic non-responsiveness may refer to a lack of functional translation, meaning that even when some physiological modulation is detectable, it does not lead to meaningful changes in symptoms, self-regulation, or clinically relevant outcomes. This distinction matters because signal change alone is not equivalent to therapeutic gain. The literature on HRV biofeedback contains both evidence of benefit and evidence of uneven or null effects in some contexts, which supports the need to separate physiological responsiveness from clinically meaningful responsiveness [4].

Third, autonomic non-responsiveness may be transient, contextual, or persistent. In some individuals, it may reflect a temporary state shaped by factors such as acute stress, fatigue, poor sleep, intercurrent illness, inflammation, pain, or transient autonomic overload. In others, it may represent a more stable limitation related to low resting vagal tone, reduced autonomic reserve, impaired baroreflex function, dysfunctional breathing, disease burden, or a mismatch between the intervention and the individual’s physiological capacity at that moment. The available literature already supports pieces of this interpretation by pointing to responder characteristics and breathing-related limitations [23,24].

**Figure 1:**
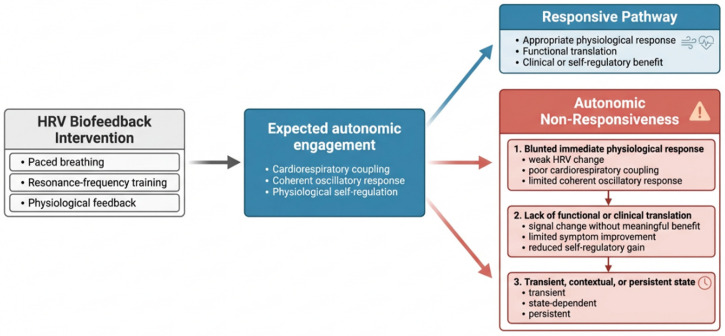
Conceptual framework of autonomic non-responsiveness during HRV biofeedback. The figure provides a schematic and hypothesis-generating representation of the proposed construct.

This conceptual lens allows non-response to be interpreted not merely as a failure of technique or adherence, but as a potentially informative state at the intersection of physiology, context, and intervention design. Our framing also creates the conceptual bridge to the next question: why HRV biofeedback may fail in some individuals and how future, more adaptive approaches may respond to that variability more intelligently [24] (Figure 1).

## 4. Why Might HRV Biofeedback Fail?

Failure of HRV biofeedback should not be interpreted as a simple matter of poor adherence or insufficient motivation. In many cases, an attenuated or absent response could reflect an interaction between state physiology and personalization failure. The intervention may be applied correctly, but the organism may not be in a condition that allows meaningful autonomic engagement, or the protocol may not be sufficiently matched to the individual’s physiological profile at that moment. This distinction is important, because it shifts the discussion from generic “noncompliance” toward a more clinically useful model of constrained responsiveness [8,11,25].

### 4.1. Physiological Factors

At the physiological level, some individuals may have a reduced capacity to generate the oscillatory autonomic response on which HRV biofeedback depends. The intervention relies in part on amplifying respiratory sinus arrhythmia and strengthening baroreflex-mediated heart period oscillations. These effects are likely to be diminished when autonomic flexibility is limited, baroreflex function is impaired, or resting vagal reserve is already low [3,19]. Such constraints may be particularly relevant in patients with substantial disease burden, chronic autonomic dysregulation, cardiometabolic disease, chronic kidney disease, or longstanding stress-related physiological state, all of which have been associated with lower HRV and diminished adaptive reserve [14,15,16,17].

Additional physiological barriers may be transient but still clinically important. Fatigue, poor sleep, pain, inflammatory activation, and intercurrent illness may all shift autonomic balance in a direction that makes resonance-based engagement less likely or less stable [26,27,28,29]. Drugs that alter heart rate, conduction, respiratory pattern, alertness, or autonomic tone may influence both baseline HRV and the apparent response to training [1,10]. Moreover, in the presence of ectopic beats, frequent artifacts, or non-sinus rhythms, HRV becomes harder to interpret, and the absence of an expected response may reflect signal limitations rather than true biological non-responsiveness [1,10].

Inflammatory activity may represent another relevant physiological factor. Elevated inflammatory markers are closely linked to autonomic dysregulation, including reduced vagally mediated HRV and persistence of sympathetic predominance. In this context, low responsiveness to HRV biofeedback may reflect a state in which an inflammatory state narrows the physiological capacity for vagal recruitment, baroreflex engagement, or recovery-related autonomic adaptation. This interpretation is supported by studies showing that baseline inflammatory status may influence the acute autonomic and neurophysiological response to HRV biofeedback, with higher TNF-α levels being associated with minimal training effectiveness and persistent sympathicotonic patterns [30,31].

Structural and respiratory particularities should also be considered when interpreting low responsiveness. Patients with anatomical airway defects, chronic tracheostomy, previous prolonged mechanical ventilation, or altered upper-airway mechanics may have limited ability to generate stable respiratory patterns during feedback-based training [32,33].

Neurodegenerative disorders may represent another context in which reduced responsiveness requires specific attention. In Parkinson’s disease, autonomic dysfunction, impaired respiratory control, altered sensorimotor integration, and disturbed cardiorespiratory coupling may limit the ability to generate or sustain stable voluntary modulation of physiological signals during feedback-based training. In this setting, low responsiveness may reflect disease-related limitations in heart-brain and cardiorespiratory synchronization rather than protocol inadequacy alone [34].

Moreover, environmental conditions, including cold exposure and climatic stressors, may also influence autonomic tone, recovery patterns, and HRV biofeedback responsiveness, particularly in adolescents or populations living in cold-climate regions [30].

### 4.2. State-Dependent Factors

Even when baseline physiology is not severely constrained, responsiveness may vary according to the individual’s immediate state. Acute stress, hypervigilance, anticipatory anxiety, pain, or sympathetic overactivation may interfere with the transition into a regulated breathing-autonomic pattern. HRV is known to be sensitive to psychological stress and affective arousal, and these states may reduce the likelihood that a person can enter or sustain the cardiorespiratory conditions required for effective training [17,26]. In practice, this means that a patient may appear to be a poor responder during one session, yet respond more favorably under different contextual conditions.

A limited autonomic response during a period of acute burden could reflect a temporarily narrowed physiological window for adaptation. For this reason, autonomic non-responsiveness may sometimes be better understood as a fluctuating condition rather than a stable characteristic of the individual.

### 4.3. Methodological Factors

A second major explanation lies in the intervention itself. HRV biofeedback is often presented as a coherent technique, yet the literature shows marked variability in how it is practiced, including differences in resonance-frequency assessment, pacing strategy, session structure, respiratory monitoring, and outcome selection [7,29]. If the individual resonance frequency is not properly identified, if a fixed breathing rate is applied indiscriminately, or if sessions are too brief to produce stable engagement, the intervention may underperform for reasons that are methodological rather than physiological [7,29].

Measurement choices can also contribute to apparent failure. Suboptimal devices, poor signal quality, insufficient respiratory monitoring, or inappropriate reliance on metrics that do not reflect the intended mechanism may all obscure the true response or falsely suggest non-response [10,29].

### 4.4. Cognitive and Behavioral Factors

Finally, cognitive and behavioral factors should not be ignored, although they should not be treated as the only explanation. Effective HRV biofeedback requires active learning and sustained attention. Reduced engagement, difficulties in understanding the technique, unrealistic expectations, or limited interoceptive awareness may limit training effects, especially early in the learning process [24,35]. Dysfunctional breathing patterns may also be relevant, because they may represent both a physiological and a learned behavioral barrier to achieving the target autonomic pattern [23].

**Figure 2:**
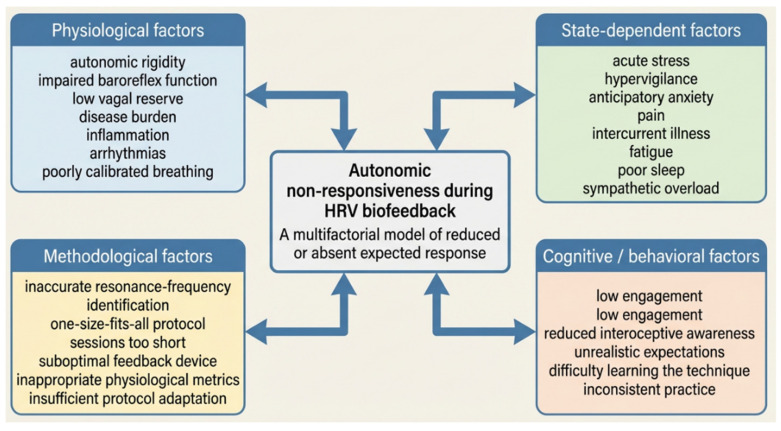
Why HRV biofeedback may fail: determinants of non-responsiveness. The categories are separated for conceptual clarity, but overlap may occur in practice.

Therefore, these observations support a more nuanced interpretation of non-response. Apparent failure may arise from person–protocol mismatch, inadequate physiological readiness, or incomplete skill acquisition, rather than from a simple unwillingness to participate (Figure 2).

## 5. Clinical Implications of Autonomic Non-Responsiveness

Autonomic non-responsiveness matters clinically because failure to benefit from HRV biofeedback may otherwise be interpreted too narrowly. A weak or absent response may be mislabeled as poor adherence, inadequate technique, or simple treatment inefficacy, even when the underlying issue is more complex and may involve state-dependent physiology, breathing-related limitations, or a mismatch between the individual and the protocol used. This distinction is particularly important in a field where both therapeutic promise and substantial methodological heterogeneity coexist, and where individualized resonance-frequency assessment, procedural calibration, and breathing pattern evaluation may meaningfully shape the observed response [4,29,36,37].

A second implication is therapeutic. If non-response is treated as definitive evidence that HRV biofeedback is ineffective for a given patient, a potentially useful intervention may be abandoned too early. Yet the available literature suggests that HRV biofeedback can be associated with improvements in autonomic function, symptoms, stress-related cardiovascular reactivity, and in some settings even longer-term cardiovascular outcomes, while review data in chronic disease support its broader clinical feasibility. From this perspective, apparent failure may sometimes signal the need for reassessment and adjustment rather than discontinuation. The more relevant clinical question may therefore be not simply whether HRV biofeedback works, but under what physiological and procedural conditions a given patient is able to respond meaningfully [21,38,39,40,41,42].

A third implication concerns interpretation of the response itself. In some cases, absent or attenuated benefit may be informative rather than merely disappointing: it may point to reduced autonomic adaptability, limited physiological readiness, or diminished autonomic reserve. Although autonomic reserve is not yet standardized as a universally accepted operational construct in HRV biofeedback research, related concepts such as vagal or parasympathetic reserve have been described in previous biofeedback literature [43]. Used cautiously, this background concept helps frame autonomic non-responsiveness not simply as treatment failure, but as a possible marker of constrained regulatory capacity and increased vulnerability. Seen in this light, recognition of non-response becomes clinically valuable in its own right, because it may help identify patients who require more individualized, staged, or adaptive forms of autonomic intervention [44,45].

## 6. From Fixed Protocols to Adaptive Closed-Loop Biofeedback

Current HRV biofeedback practice often remains relatively static in its operational logic. In many applications, participants are trained using a fixed target breathing rate, a previously determined resonance frequency, or a standardized feedback structure that is maintained across sessions. Yet recent methodological work shows substantial heterogeneity in how HRV biofeedback is delivered, including differences in resonance-frequency assessment, pacing strategy, session structure, and outcome reporting. Moreover, resonance frequency may not remain fully stable over time, suggesting that a protocol that is appropriate at one moment may not be equally appropriate later. Taken together, these observations raise an important clinical question: whether at least some cases of apparent non-response reflect not only patient-related limitations, but also the limitations of relatively fixed intervention formats [7,29,36].

From this perspective, the next conceptual step is not necessarily a more complex protocol, but a more responsive one. An adaptive closed-loop approach would treat HRV biofeedback as an ongoing regulatory process in which physiological signals are continuously measured, the incoming response is interpreted, the intervention is adjusted, and the result is then re-evaluated.

**Figure 3:**
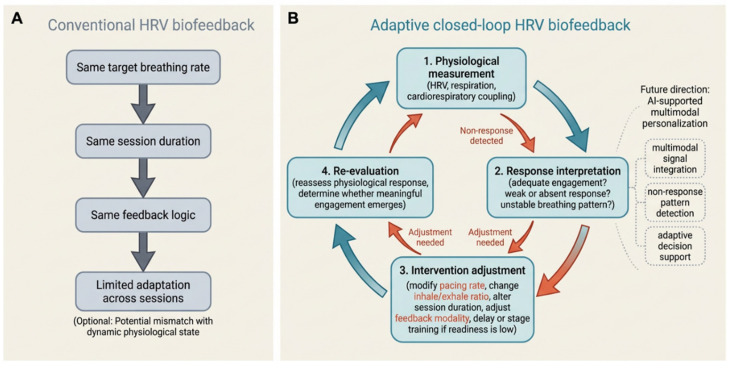
From fixed HRV biofeedback to adaptive closed-loop systems. This figure illustrates a future-oriented conceptual model.

In practical terms, such a loop could involve real-time monitoring of cardiorespiratory coupling, oscillatory coherence, breathing pattern quality, or other markers of physiological engagement, followed by modification of pacing rate, inhalation–exhalation ratio, training duration, or feedback modality when the desired response fails to emerge. This logic is consistent with broader control-systems thinking in biofeedback and with other psychophysiological feedback fields, where online measurement and feedback are understood as core features of self-regulatory learning [42,46] (Figure 3).

Importantly, this section is intended as a conceptual bridge rather than a description of a mature or clinically validated HRV biofeedback platform. Although engineering-oriented and proof-of-concept work has already explored explicit closed-loop approaches to HRV modulation, including adaptive control models and closed-loop systems designed to influence HRV in real time, such efforts remain preliminary and should not be mistaken for established clinical solutions.

The main point here is modest: if autonomic responsiveness is dynamic, state-dependent, and individually variable, then future HRV biofeedback systems may need to become correspondingly dynamic, state-sensitive, and individualized in how they deliver feedback [7,47,48].

## 7. A Conceptual Role for Artificial Intelligence

At present, the role of artificial intelligence (AI) in this context is best understood as prospective rather than clinically established. If future HRV biofeedback systems are expected to move beyond relatively fixed delivery formats, AI may become useful not because it replaces physiological reasoning, but because it can help manage complexity.

In principle, AI-based approaches could integrate multimodal streams of information—such as HRV-derived features, respiratory patterns, heart rate, movement, sleep-related data, contextual variables, or other wearable signals—and extract patterns that are difficult to recognize through single-parameter monitoring alone. This possibility is consistent with recent reviews showing that AI can enhance physiological signal analysis, support multimodal data fusion, and strengthen personalized wearable monitoring in settings where human physiology is dynamic, nonlinear, and highly individualized [49,50].

Within such a framework, AI might be especially relevant for identifying patterns of non-response. Rather than treating absent benefit as a uniform category, future systems could be trained to detect whether a given session reflects poor physiological engagement, unstable breathing behavior, transient overload, or a more persistent low-responsiveness profile.

Related work outside HRV biofeedback already shows that machine-learning approaches can classify stress-related physiological states from wearable data, support passive and ambulatory monitoring of fluctuating mental states, and use multimodal personalized models to detect individual-specific patterns over time. Conceptually, this makes AI relevant not because it proves the existence of autonomic non-responsiveness, but because it may eventually help distinguish transient from persistent forms of reduced responsiveness and place them in a more individualized physiological context [51,52,53,54].

A further potential contribution of AI would be decision support for personalization. If a system can detect that the expected response is not emerging, it could in principle recommend adjustments in pacing rate, inhalation–exhalation ratio, session duration, feedback intensity, timing of practice, or even the need to defer training under conditions of poor physiological readiness.

Importantly, this article does not present such a system, nor does it claim that a clinically validated AI framework for autonomic non-responsiveness already exists. The point is more limited and conceptual: if future HRV biofeedback is to become more adaptive, then AI may offer one possible means of integrating multimodal information, recognizing non-response patterns, and supporting more individualized intervention logic [49,50,54].

Moreover, ethical and clinical safeguards are essential if AI is used to support adaptive HRV biofeedback. AI should be considered a decision-support tool rather than an autonomous decision-maker for prescribing, approving, or modifying biofeedback protocols. Final responsibility for protocol selection, supervision, approval, and modification should remain with the treating physician or an appropriately trained healthcare professional. If an AI-generated recommendation proves inappropriate or harmful, responsibility should be assessed according to the source of failure: the clinician is responsible for contextual interpretation and safe clinical use; the healthcare institution is responsible for governance, credentialing, implementation, monitoring, and documentation; and the developer or vendor is responsible for technical validity, transparency, cybersecurity, post-market surveillance, and accurate communication of intended use, limitations, and foreseeable risks. Therefore, any AI-assisted biofeedback system should include predefined safety boundaries, explainable outputs, audit trails, human override, and escalation rules that prevent unsupervised autonomous protocol adaptation [55,56,57].

## 8. Research Agenda

### 8.1. How Should Autonomic Non-Responsiveness Be Operationally Defined?

A first priority is operational definition. If autonomic non-responsiveness is to become more than a descriptive label, future studies will need to define it prospectively rather than retrospectively.

At minimum, such a definition should specify: (1) the expected physiological response during HRV biofeedback, (2) the extent to which that response must be absent or attenuated, (3) whether lack of response must persist across repeated sessions, and (4) whether the intervention was delivered under methodologically adequate conditions, including appropriate resonance-frequency assessment and breathing guidance. This is especially important in a field where HRV biofeedback protocols remain heterogeneous and where both breathing-related limitations and responder characteristics may influence observed efficacy [7,23,24,29].

At this stage, we do not propose fixed numerical thresholds, because these would be premature without prospective validation. Rather, we suggest that future studies should test candidate operational criteria across physiological, procedural, and clinical domains.

Future studies should incorporate methodological features that allow true physiological low responsiveness to be distinguished from protocol variability, measurement limitations, and nonspecific intervention effects. When clinical outcomes are assessed, sham biofeedback or active-control conditions and blinded outcome assessment should be considered to reduce expectation effects and interpretation bias.

### 8.2. Which Biomarkers or Signal Patterns Best Identify It?

A second question concerns identification. Future work should determine which combination of biomarkers or physiological signal patterns best captures non-responsiveness during HRV biofeedback. Standard HRV indices will likely remain relevant, but they may be insufficient on their own.

Candidate markers may need to include respiratory rate and stability, cardiorespiratory synchrony, oscillatory coherence, baroreflex-related response, and the consistency with which these signals change under paced breathing near resonance. In practice, the most useful marker set may prove to be multimodal rather than single-parameter, combining HRV metrics with respiration and other features of physiological engagement [19,29,58,59].

### 8.3. When Is Non-Responsiveness Transient, and When Does It Reflect Deeper Autonomic Dysfunction?

A third research priority is temporal interpretation. Not all non-response is likely to have the same meaning. In some cases, reduced responsiveness may be transient and state-dependent, shaped by acute stress, fatigue, poor sleep, medication effects, breathing instability, or temporary autonomic overload. In others, it may reflect a more persistent limitation in autonomic adaptability or regulatory capacity.

Distinguishing between these possibilities will require repeated measurements across sessions, attention to the many factors known to influence HRV, and explicit study designs that separate momentary physiological state from more stable autonomic vulnerability. This question is central if autonomic non-responsiveness is to be interpreted not simply as treatment failure, but as a potentially informative marker of constrained physiological readiness [24,44,60,61].

### 8.4. What Is the Minimal Adaptive System Needed to Test This Concept Without Overextending into Speculative AI?

A fourth question is methodological and translational: what is the simplest adaptive system that could meaningfully test this concept? A reasonable starting point would not require sophisticated artificial intelligence. Instead, an initial proof-of-concept system could combine wearable or real-time HRV monitoring with respiration tracking and a limited set of rule-based adaptations—for example, adjusting pacing rate, cueing strategy, or session duration when expected physiological engagement does not emerge.

Only after such minimal adaptive systems demonstrate feasibility and interpretable benefit would it be justified to move toward more complex algorithmic or AI-assisted approaches. In this sense, the immediate research goal is not to build a fully intelligent platform, but to establish whether a modest closed-loop, state-sensitive HRV biofeedback system can detect and respond to non-response in a clinically meaningful way [47,62,63,64].

A stepwise validation pathway is therefore needed. Initial studies should define reproducible physiological response criteria during HRV biofeedback, including HRV indices, respiratory stability, cardiorespiratory coupling, oscillatory coherence, and baroreflex-related markers. These criteria should then be tested across repeated sessions to determine whether low responsiveness is transient and state-dependent or persistent and reproducible. Subsequent studies should examine whether these physiological patterns predict clinically meaningful outcomes, such as symptom improvement, functional change, blood pressure response, stress regulation, or disease-specific endpoints.

## 9. Conclusions

HRV biofeedback remains a promising non-pharmacological approach for influencing autonomic regulation, yet its effects are clearly not uniform across individuals or clinical states. The present conceptual framework suggests that part of this variability may reflect a proposed state of autonomic non-responsiveness, in which expected physiological engagement is attenuated, absent, or insufficiently translated into meaningful clinical benefit. Framing non-response in this way helps move the discussion beyond simplistic explanations based solely on adherence or technique and toward a more clinically relevant understanding centered on physiological readiness, contextual state, and person–protocol fit. In this context, future adaptive and possibly AI-assisted systems should be viewed not as replacements for physiological reasoning, but as potential tools for detecting heterogeneous response patterns and for guiding more responsive, state-sensitive forms of intervention.

However, the clinical meaning of autonomic non-responsiveness remains unproven. At present, it should be viewed as a candidate signal of constrained autonomic adaptability rather than as a validated marker of autonomic reserve or physiological vulnerability. Future prospective studies are needed to determine whether attenuated HRV biofeedback responsiveness has reproducible associations with autonomic function, disease severity, clinical outcomes, or treatment response. Ultimately, clarifying and testing the concept of autonomic non-responsiveness may help refine patient selection, strengthen intervention design, and advance a more precise model of autonomic biofeedback.

## Data Availability

No new datasets were generated or analyzed during the preparation of this conceptual review.

## Abbreviations

The following abbreviations are used in this manuscript:

AI	Artificial intelligence
HRV	Heart rate variability
LF	Low frequency
HF	High frequency
VLF	Very low frequency
LF/HF	Low-frequency/high-frequency ratio
RSA	Respiratory sinus arrhythmia
Hz	Hertz
